# A One Health approach to assessing occupational exposure to antimicrobial resistance in Thailand: The FarmResist project

**DOI:** 10.1371/journal.pone.0245250

**Published:** 2021-01-28

**Authors:** Duangdao Sudatip, Kittipong Chasiri, Anamika Kritiyakan, Wantanee Phanprasit, Chuanphot Thinphovong, Surapee Tiengrim, Visanu Thamlikitkul, Rim Abdallah, Sophie Alexandra Baron, Jean-Marc Rolain, Serge Morand, Markus Hilty, Anne Oppliger

**Affiliations:** 1 Faculty of Public Health, Department of Occupational Health and Safety, Mahidol University, Bangkok, Thailand; 2 Institute for Infectious Diseases, University of Bern, Bern, Switzerland; 3 Department of Occupational Health and Environment, Unisante, University of Lausanne, Lausanne, Switzerland; 4 Faculty of Tropical Medicine, Mahidol University, Bangkok, Thailand; 5 Faculty of Veterinary Technology, Kasetsart University, Bangkok, Thailand; 6 Faculty of Medical Technology, Department of Clinical Microbiology and Applied Technology, Mahidol University, Nakhon Pathom, Thailand; 7 Faculty of Medicine, Division of Infectious Diseases and Tropical Medicine, Department of Medicine, Siriraj Hospital, Mahidol University, Bangkok, Thailand; 8 MEPHI, IHU Méditerranée Infection, Aix-Marseille University, Marseille, France; 9 Institut des Sciences de l’Evolution, CNRS, Université de Montpellier, Montpellier, France; Nitte University, INDIA

## Abstract

This Southeast Asia-Europe research project will use a One Health approach to identify the major parameters responsible for the presence of animal-associated antimicrobial resistant bacteria in animal production facilities in Thailand and the risk of their transmission from animals to humans. We will focus on traditional, small, extensive pig and poultry farms where information on antibiotic use is scarce and animals live in close contact with humans. This cross-sectional study will be based on the epidemiological analysis of the antimicrobial resistance (AMR) present in fecal samples from animals and humans. Extended spectrum beta-lactamase producing Enterobacteriaceae (ESBL-E) and Enterobacteriaceae resistant to colistin will be actively searched in the feces of farm animals (pigs and poultry), small wild rodents and farmers. Phenotypic (selective plating) and genotypic (multilocus seuquence typing and sequencing) methods will be used for the detection of AMR, the identification of antibiotic resistance genes (ARGs) and the characterization of strains carrying resistance genes. Questionnaires will be administered to investigate the effects of antibiotic use, farm characteristics and biosecurity measures on the occurrence of AMR in animals. Subsequently, the fecal carriage of AMR and ARGs in farmers will be compared to a control population with no occupational contacts with animals, thus enabling an estimation of the risk of transmission of AMR/ARGs from animals to farmers.

## Introduction

One Health is a collaborative, multi-sectoral, trans-disciplinary approach working at local, regional, national, and global levels used to achieve optimal health and well-being outcomes which recognize the interconnections between people, animals, plants and their shared environment (www.onehealthcommission.org). The One Health approach recognizes that people’s health is closely connected to the health of animals and our shared environment.

The approach is particularly relevant for combating antimicrobial resistance (AMR) since animals and humans are colonized by the same bacteria species and cured with the same classes of antibiotics. Thus, the presence of ARGs in animals can have a significant impact on public health if they are transmitted to clinically relevant bacteria able to infect humans [1].

Southeast Asia (SEA) is considered a hot spot for AMR [2], and it is also a region with an important food animal production sector.

The poultry sector is the largest agribusiness in Thailand, making up about 52% of total national meat production [3]. Three types of production models coexist: smallholder backyard farming with although non-commercial provides an important source of income from selling chickens to local markets (1–99 birds); medium-scale or semi-industrial farming (100–10’000 birds); and large-scale industrial farming (> 10’000 birds) with more than 50% of production for export. Most farms are backyard farms (> 90% of the total). It has been estimated that an overall average of 74.4 mg of in-feed antimicrobials was used to raise every 1kg of live chicken in SEA [4].

Pig production is also significantly present in Thailand. A 2011 FAO report stated that pig production there had developed an intensive industry with many medium (501–5000 heads:18.6% of farms) to large-scale (> 5000 heads. 1.44% of farms) producers, although traditional, small, extensive family farms (1–50 pigs) were still the majority (80% of farms). These small-holders raise indigenous pig breeds for personal consumption and as a supplementary source of income. In 2010, 8.3 million pigs were produced and it has been estimated that an overall average of 286.6 mg of in-feed antimicrobials (including colistin) were used to raise every 1kg of live pig in SEA [4].

Small family farms, however, are often associated with poor hygiene and low biosecurity, with few barriers to a potential contact or bacterial transmission between farm animals, humans, and wildlife [5]. Antimicrobial-resistant bacteria can be transmitted from animals to humans via direct contact or contaminated farm environment: manure, air or wild animals such as rodents [6–11]. Measures must be implemented to prevent the potential transmission of AMR from animals to farmers. This requires the identification of factors favoring the selection and dissemination of resistant strains of bacteria on farms.

Today, the two biggest worldwide concerns involving resistance to antimicrobials are the increase in extended-spectrum beta-lactamase producing Enterobacteriaceae (ESBL-E) [12,13] and the emergence and rapid dissemination of plasmids carrying the *mcr-1* gene, which confers resistance to colistin (polymyxin E) [14,15]. These plasmids were first identified in 2014, in pigs in China [15] and were then reported in clinical samples from other countries [16]. In 2012, it was shown that 6% of healthy Thai individuals were carriers of colistin-resistant *Klebsiella pneumoniae* [14], and it was also demonstrated that numerous human carriers of colistin- resistant bacteria had not previously received colistin therapy [17]. Colistin has been extensively used in animal pig production, often as a growth promoter [4,18]. Recently, a 19–22% prevalence of the *mcr-1* gene was reported in 180 strains of *Escherichia coli* isolated from pig and chicken farms in Vietnam [19] and clonal transmission of colistin-resistant *E*. *coli* from a domesticated pig to a human was demonstrated in Laos [17].

Concerning ESBL-E, Thai data from 2005–2007 showed a very high prevalence in different clinical specimens, with 30–40% of *E*. *coli* and 27–39% of *K*. *pneumoniae* isolates [13]. Moreover, this high prevalence of antibiotic resistant bacteria involved not only pathogenic clinical strains but also commensal bacteria. For example, 58% of asymptomatic volunteers of a rural region showed fecal carriage of ESBL-E and CTX-M producers [20]. The inappropriate use of antibiotics in human has been suggested as the cause of this alarming prevalence in the community, however, clearly significant associations between the presence of ESBL-E and antibiotic use is missing [21,22]. Other factors could also be responsible: occupational contact with pigs has been suggested as a risk factor for acquiring ESBL-E in the Netherlands [10]. It was also reported that 77.3% of the *E*.*coli* isolated from 30 healthy farm workers were ESBL producers, suggesting a potential zoonotic transmission from animal to farmers [23].

Despite the alarming carriage of ESBL-E and colistin-resistant bacteria in the community and the significant use of antibiotics in pig and poultry farming, data concerning antimicrobial usage (AMU) and the prevalence of AMR in pig and poultry farm environments are poorly documented. To fill this gap, in 2016, the Thai cabinet endorsed a National Strategic Plan on Antimicrobial Resistance 2017–2021. This plan is Thailand’s first national strategy specifically targeted AMR with an emphasis on multi-sectoral collaboration through the One Health approach. The strategy considers both national and international policies in order to systematize actions to address AMR [24]. The National plan is also aligned with the Global Action Plan, reflecting the country’s commitment to join forces internationally to resolve AMR issues. The FarmResist project will operate with the goals of the National Plan: it will provide new data and new ways to prevent the occupational exposure risks facing the traditional small extensive family farmers who run 80–90% of Thailand’s farms.

## FarmResist project design

### Study objectives

The objectives are: 1) to assess the occupational risk of pig and poultry farmers being colonized by animal-associated ESBL-E and colistin-resistant Enterobacteriaceae and 2) to look at the associations between farm characteristics, included the presence of rodents carriers of AMR and the prevalence of AMR in the farm’s animals and the farmers.

### Methods

#### Human ethical approval

**Fig 1:**
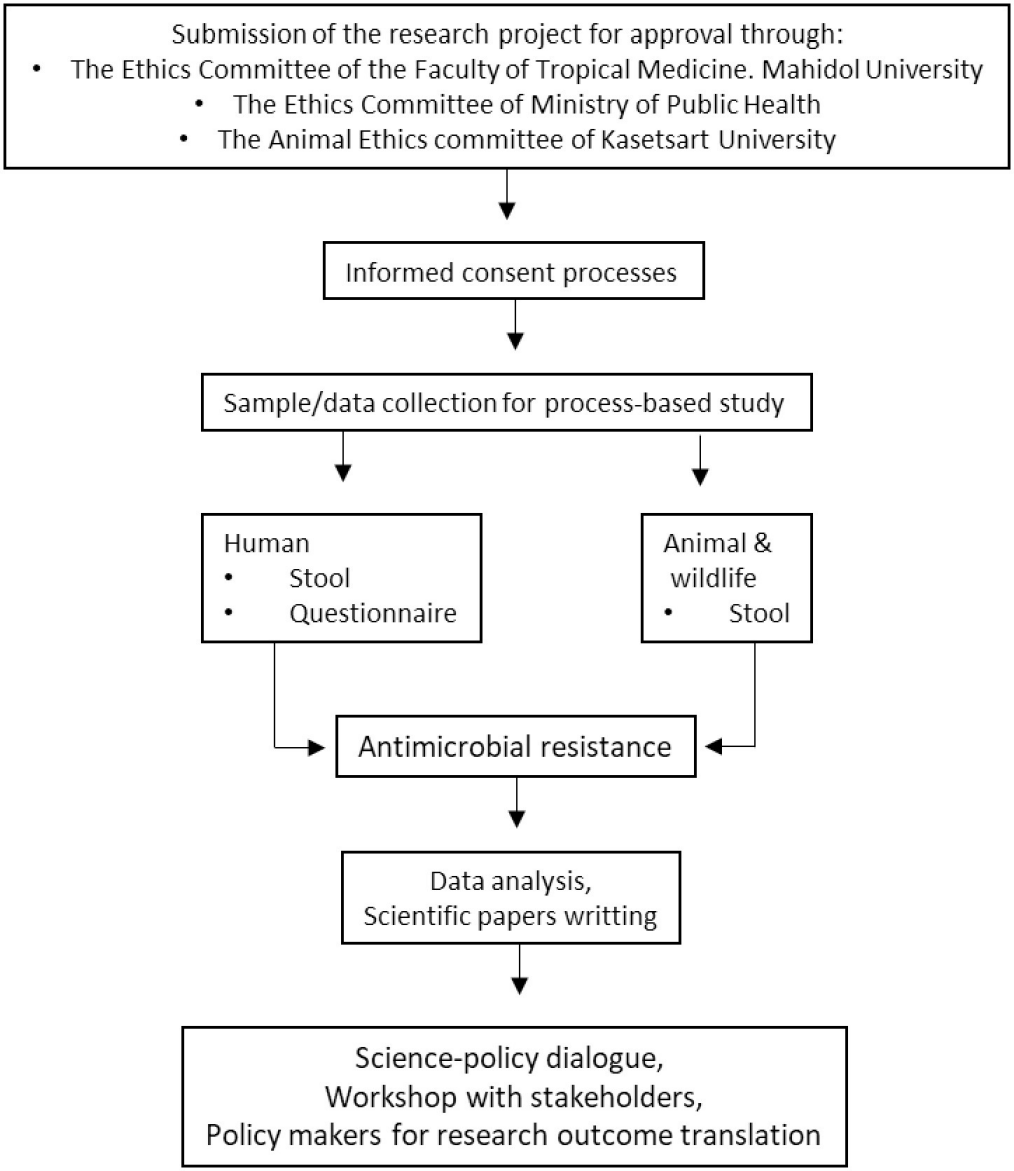
Schematic diagram of study design.

Mahidol University’s Ethics Committee for the Faculty of Tropical Medicine approved this research protocol involving the collection of biological specimens and data from humans (Certification number: MUTM-2018-035-01). Because they are sharing the same study site in Thailand’s Nan Province, approval for the FarmResist project also served as approval for a project entitled: Predictive scenarios of health in Southeast Asia: linking land use and climate changes to infectious diseases (FutureHealthSEA). In terms of research workflow, an overview of the research is presented in the schematic diagram below (Fig 1). This includes pre-research activities (i.e., document preparation for ethical, informed consent processes), samples and data collection (human, pig, poultry and rodent), data analyses, database construction and post-research actions, i.e., research translation communicating findings and outcomes to local administrations (local public health offices and livestock offices) and participants (farm workers and farm owners) through a meeting or workshop.

#### Animal ethical approval

Kasetsart University‘s Scientific Research Committee gave ethical approval for the animal parts (ACKU 62-VTN-010) of this study. The animal protocol covers the study in pigs, chickens and rodents rectal swab samples to be collected in Nan Province. This will involve working with livestock volunteers (at the village level) and officers from Thawangpha District’s livestock department to explain the study’s objectives and the methods used for biological sample collection to local farm owners.

#### Knowledge, attitudes and practices about antibiotics use

Understanding farmers’ knowledge and practices with regards to antibiotics use can help to identify their knowledge gaps, behavioral patterns, and other barriers contributing to the misuse of antibiotics. Ultimately, it will help to improve antibiotic management at the interface between human and animal health. We will perform a “Knowledge, Attitudes and Practices” (KAP) survey to quantify and qualify the knowledge (a set of understandings), attitudes (positions toward), and practices (behaviors and actions) of antibiotic uses among participants. This will take form of questionnaires and statistical processing of the collected information [25]. The participants will be the farmers involved in the FarmResist project, local public health, animal health officers, and village health and livestock volunteers who are responsible for community and livestock health in village. KAP survey are useful tools used in public health to identify effective strategies for moving towards safer practices [26]. Based on KAP information and antimicrobial analyses, a workshop will be conducted with local administrators (livestock department, public health department), farmers involved in the study, and local village health and livestock volunteers. Thai social scientists will be involved in the preparation of both the KAP survey and the workshop.

#### Study sites and study population

Twelve villages from Nan Province will be selected to produce varied environmental gradient, *i*.*e*., 4 villages in landscapes with high forest cover, 4 villages with intensive agricultural activity and 4 villages in more urbanized settings.

The study’s target population will be male or female individuals between 16–70 years old, who raise poultry and/or pigs as full-time or part-time occupation and who reside in one of the 12 villages selected. Participants with no contact with farm animals will be selected for the control group in the same villages than farmers and we will insure to have a representative group in term of age, sex and economic status. The selection criteria include no problems communicating in Thai and voluntarily signing the Informed Consent/Assent Form. Participant recruitment will be done with the help of local communities (at the village level). The investigation team will conduct meetings with local public health officers, local veterinary teams, heads of sub-districts, village chiefs, and local health volunteers in order to co-design the plans for human and animal sample collection.

#### Sampling

**Fig 2:**
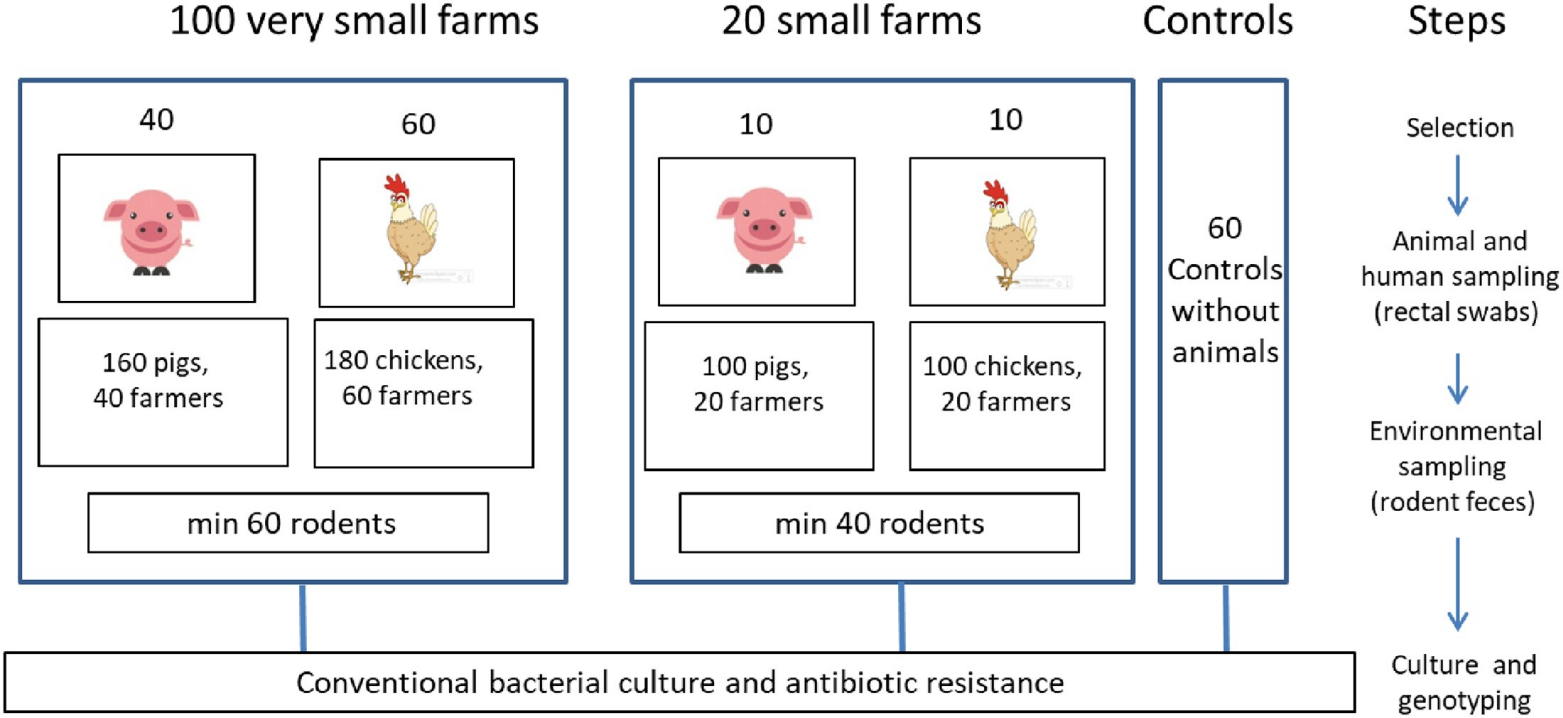
Schematic diagram of the sampling strategy.

Sampling will be performed as illustrated in Fig 2. A selection of pig and chicken farms will be chosen in collaboration with the local authorities (local public health offices and livestock offices and owners will be contacted for their consent. The selection will include: 20 small farms (10 pig and 10 poultry) and 100 very small farms (40 with pigs only, 60 with chickens only). Very small farms are these housing between 1–20 animals and small farms, between 20–50 animals.

Each participating farm will be visited by a team of skilled investigators and questionnaire will be administered to the participants. The questionnaire will include: basic personal information (age, address, occupation and educational level); socioeconomic information; risk factors for infectious diseases; knowledge and attitudes about diseases and health; history of antibiotic use in medical treatments and for agrichemical purposes; and work habits, biosecurity measures, and risk factors for AMR carriage.

Rectal swabs will be collected from 1–10 adult pigs or chickens per farm. We will take a maximum of 3 and 4 animals in, respectively, very small chicken and very small pig farms and maximum 10 animals per small farm. Therefore, we expect to sample 20% of the total number of animal per farm. At the same time, between 10–20 custom-made rodent-traps (which keep the animal alive) will be placed around and inside the farm. The day after, the traps will be checked for the presence of rodents and animals will be sacrificed, fecal samples collected as well as tissues for another study. Farmers and volunteers non-exposed to farm animals will be given a stool kit to be collected the next day by the investigators when they come to check the rodent traps for the presence of animals. On arrival the laboratory (within 4 hours), samples will be processed and stored (-80°C) immediately. Animal rectal swab samples will be transported on ice and stored at 4°C until analysis.

#### Bacteria species identification

To detect ESBL-E and/or colistin-resistant Enterobacteriaceae, rectal swab samples will be enriched overnight in specific Tryptic Soy broth incubated at 35 ± 2 °C The 100 μL of overnight TS broth is subcultured on 2 μg/mL ceftriaxone–supplemented MacConkey agar and 1 μg/mL colistin–supplemented MacConkey agar, incubated at 35 ± 2 °C for 24–48 hours. Bacterial identification will be performed on 2–5 colonies per plate using Matrix Assisted Laser Desorption Ionisation-Time of Flight (MALDI-TOF MS) (Bruker Daltonik) and antimicrobial susceptibility tests will be done by screening with disk diffusion method, using amoxicillin/clavulanic acid (AMC, 20/10 μg), ceftriaxone (CRO, 30 μg), ceftazidime (CAZ, 30 μg), cefepime (FEP, 10 μg), Ertapenem (ETP, 10 ug), Colistin (CT, 10 ug), and Cefoxitin (FOX, 30 ug) placed on Mueller-Hinton agar (Oxoid, Baskingstoke, England) and incubated at 35°C for 24 hrs. according to European Committee on Antimicrobial Susceptibility Testing (EUCAST) recommendations. The agar dilution method will be used to determine the minimum inhibitory concentration (MIC) for ceftriaxone, colistin, and meropenem in positive human and animal isolates. All positive isolates will be frozen at -80°C.

#### Molecular characterization of ESBL-E and colistin resistant isolates

DNA extraction will be performed for all ESBL-E and colistin resistant isolates. Using the Illumnia Novoseq platform, Whole-genome sequencing (WGS) for approximately 200 and 600 human and animal E. coli isolates, respectively, will identify the core genome multi-locus sequencing types (MLST) and ’traditional’ MLST for all isolates. The data will also allow the identification of the respective genes responsible for the antibiotic-resistant phenotype. In combination, characterization of antibiotic resistance mechanism and strain typing will allow the quantification of transmission within the pig and chicken farm setting from animal to animals but also elucidate potential zoonotic transmission. In combination with epidemiological data, this will identify risk factors for transmission e.g. antibiotic usage and size of the animal farms. We may also identify a clustering of strains according to the geographic origin of the isolates.

A high prevalence of plasmid mediated ESBL and colistin resistance is expected. However, using short read sequencing, we will only be able to define vertical but not horizontal transmission (plasmid transfer).

#### Data analysis

An integrative data set will be created, combining quantitative and qualitative parameters, i.e., AMR, domestic animal populations, agricultural inputs such as antibiotics, as well as data obtained from questionnaire. Descriptive statistics will be used to describe AMR prevalence and AMU. The association between presence of specific AMR or specific genes of resistance and other farm parameters will be calculated with generalized linear mixed models. The odds of a farm to be colonised with an AMR will be estimated by performing univariate analysis with AMU and farm parameters. All statistics will be performed by a statistician specialised in the analysis of epidemiological data. The data/knowledge base and models will, finally be exploited to produce scenarios combining predictions and story-telling about the likely impacts of strategies and measures to reduce AMR levels in both animals and farmers.

## Conclusion and perspective

The Thai swine and poultry industries represent an important economic resource to the country. Besides the numerous, very big, industrialized, concentrated animal feeding operations, where workers have to respect very strict biosecurity rules to avoid animals becoming contaminated by microorganisms, the country has many more small traditional, extensive family farms where the basic biosecurity rules are unknown. These farmers give their animals drugs to ensure rapid growth and good health. These drugs are often antimicrobial substances that can be bought without a prescription. The misuse can have dramatic effects because they may increase the selection of AMR and the dissemination of resistant bacteria into the environment.

In November 2017, the leaders of the Association of Southeast Asian Nation (ASEAN) declared their commitments to develop an ASEAN strategic plan to combat AMR. This plan encourages a “One Health approach” and will help ASEAN to move towards a program of integrated AMR surveillance. It will also promote research on the impact of AMR on the environment and agriculture, and this, in turn will guide authorities towards the development of appropriates policies and regulations and help them to prepare effective evidence-based treatment guidelines to optimize the use of antimicrobials.

The present project’s findings will not only contribute to Thailand’s public health policy, by fighting AMR at the national level, they will also encourage the country to become the leader in the implementation of ASEAN’s strategic plan.

## Supporting information

S1 File (DOCX)

S2 File (DOCX)
