# Supplementary material for: A One Health approach to assessing occupational exposure to antimicrobial resistance in Thailand: The FarmResist project
Source: PLoS One. 2021 Jan 28;16(1):e0245250. doi: 10.1371/journal.pone.0245250 (PMC7842938; doi:10.1371/journal.pone.0245250)
Supplement: S1 File — (DOCX) [file pone.0245250.s001.docx]

QUESTIONNAIRE

Farm No:………. Subject ID:……………………….. Date : ………………………..

# **Part 1 Farmer behaviour and work condition**

**General characteristic of farmer**

1. Sex : ( )male ( )female
2. Age :_________years.
3. Height : _________cm
4. Weight : _________kg
5. House No :________________________
6. Village : __________________
7. Education : ____________________________________
8. Leisure activity: ____________________________________
9. Traveled or lived in other countries during the last 12 months?

( ) Yes ( ) No

If yes, where and when: ____________________________________

1. Current Job Title: ( ) Farm owner ( ) Worker ( ) Other (Please specify)________
2. How long have you been working in this farm?: _____months_______years
3. What type of work are you involved in on the farm?: ________________________
4. Working practice

|  | Description |
| --- | --- |
|  |  |
|  |  |
|  |  |
|  |  |
|  |  |

1. Other job: ____________________________________
2. Professional history

| Years | Occupation and tasks |
| --- | --- |
|  |  |
|  |  |
|  |  |

**Work condition**

1. Type of animal in your farm: pig ( ) poultry ( ) both ( )
2. How many pigs/birds do you have?

_________sows

_________farrowed piglets

_________weaned piglets

_________boars

_________ pigs in fattening unit

_________poultry

1. How many pig or poultry per worker? :_______________________
2. On your farm, are there other animals, including pets?

( ) Yes, which other animal? _______________________ ( ) No

1. Are dogs and cats able to enter the shed? ( ) Yes ( ) No
2. How many hours do you work per week in the animal house__________ hours
3. How many hours per week do you normally work (including work outside the pig or poultry buildings)? __________ hours
4. Do you currently own any pets?

1□ Yes

2□ No

1. If yes, how many pets do you own? ______
2. What types of pets do you own? (Check all that apply):

1□ Dogs

2□ Cats

3□ Birds

4□ Other (Specify)_______________________________________

1. Do you wear personal protective equipment (PPE) at your working place?

( ) Yes, please specify type of PPE _____________________________

( ) No

1. How long you wear PPE? ( ) > 50% of working time? ( ) < 50% of working time?
2. Do you consider that the air from the animal house as being ‘dusty’?

( ) Yes, very dusty ( ) Yes, a bit dusty ( ) No

1. Do you wear a respiratory protection mask at your working place?

( ) Yes ( ) > 50% of time? ( ) < 50% of time?

( ) No

1. Do you wash your hands after using any of the above PPE? ( ) Yes ( ) No

**Medical history**

Yes No do not know

29. Do you suffer from diabetes? ( ) ( ) ( )

30. Do you suffer from kidney problems? ( ) ( ) ( )

31. Do you suffer from liver problems? ( ) ( ) ( )

32. Do you take drugs which could affect your immune system?

(anti-inflammatories corticosteroids etc.) ( ) ( ) ( )

33. Do you suffer from chronique skin diseases? ( ) ( ) ( )

(Eczema, psoriasis, etc.)

34. Do you have some allergies and/or dietary issues?

( ) Yes, which ones ? _________________________________( ) No

**During the last 6 months:**

Yes No do not know

35. Did you take any antibiotic drugs ( ) ( ) ( )

If yes : How often? ____________ Why ?_________

What kind of antibiotics?­­­­­­­­­­­­­­­­­­­ __________________

Amount of using?­­­­­­­­­­­­­­­­­­­ ___________

36. Have you worked in an health care service ( ) ( ) ( )

37. Have you been hospitalized ( ) ( ) ( )

38. Did other members of your household have gastro-intestinal

/problems ( ) ( ) ( )

39. Did you suffer from gastro-intestinal ( ) ( ) ( )

40. Have you gone to the hospital because of an animal-related injury? ( ) ( ) ( )

#### **Smoking status:**

41. Are you smoking?

( ) Yes

( ) No

42. How many packages of cigarettes are you smoking each day approximately?

______ no. of cigarettes/day

Do you smoke in the animal house?;

if yes: do you wash your hands before smoking?

□ Yes, always

□ Yes, sometimes

□ Yes, rarely

□ No, never

43. Have you smoked previously (but are currently an non-smoker) ?

( ) Yes

( ) No

44. How many packages of cigarettes did you smoke each day approximately?

______ no. of cigarettes/day

_________ no. of packages of cigarettes per day

45. Since when you stopped smoking? _________ years ago

#### **Diet**

46. What diet he/she usually follows?

( ) Vegetarian ( ) Vegan ( ) Rawist ( ) Omnivore (normal)

47. What sources of meat do you usually eat?

( ) Market ( ) Hunting ( ) Your own animal

( ) Other, please specify_________

48. What sources of vegetable do you usually eat?

( ) Market ( ) Your own vegetable, Do you plant near your animal farm? ___ ( ) Other, please specify_________

49. What sources of water do you usually drink?

( ) Tap water ( ) Groundwater ( ) River

( ) Other, please specify_________

**Personal hygiene**

1. Do you wash your hands *before* eating?

1□ Yes, always

2□ Yes, sometimes

3□ Yes, rarely

4□ No, never

1. Do you wash your hands *after* eating?

1□ Yes, always

2□ Yes, sometimes

3□ Yes, rarely

4□ No, never

1. Do you sometime eat/drink in the animal house?

1□ Yes

2□ No

1. Do you have specific (dedicated) clothes for work?

1□ Yes

2□ No

a. If yes, do you remove it before to enter your home?

1□ Yes

2□ No

1. Do you take a shower after the work day?

1□ Yes

2□ No

**Contact with other animal species**

1. Have you come in contact with any wildlife animals in the past 12 months?

1□ Yes

2□ No

a. If yes, please list them: _________________________________________________

1. Outside the farm, in the past 12 months from today, have you been in direct contact with any of the following *live* animals? (Check all that apply):

1□ Chicken

2□ Pigs

3□ Rodent

4□ Other, please specify_________________

**Part 2 Farm characteristic**

1. Type of farming:( ) maternity;( ) fattening unit;( ) laying hens ;( ) chicken meat
2. Size of farm :( ) small ;( ) medium
3. For fattening unit, do weaning piglets come from one producer or several?

( ) one ( ) several

1. For maternity, does sow come from own breeding or external producer?

( ) own breeding ( ) external producer

1. For maternity, does sow come from same village or another village?

( ) same village ( ) another village

1. Does the animal house open or close system?

( ) open ( ) close

1. What type of food do animal eat?

( )Animal food ( ) Human food ;( ) Other, please specify _____________

1. Do you have all in – all out system?

( )Yes ( ) No

1. Do you have a rodent eviction?

( )Yes ( ) No

1. How do you manage waste water in your farm : ______________________________________________________________________________________________________________________________________________________
2. All farm sections are cleaned and disinfected?

( )Yes, If yes at which frequency? ______________________

( ) No

1. All farm sections are disinfected?

( )Yes, If yes at which frequency? ______________________

( ) No

1. After cleaning/disinfection of sections, is there a dry period of at least 24 hours?

( )Yes ( ) No

1. List all the products used for cleaning and disinfection:

| No. | Products |
| --- | --- |
|  |  |
|  |  |
|  |  |
|  |  |
|  |  |

1. Does the farm have presence of foot bath?

( )Yes, do you usually use it? :( ) yes ( ) no

( ) No

1. What is the name of the disinfectant for footbath__________dilution _____________
2. What is the name of the disinfectant for for surfaces: ______dilution______________
3. How frequently do you change the footbath? _______________________________
4. Do you change frequently of disinfectant type (name)?

( )Yes, If yes how many?______________________

( ) No

Describe in detail the process of surface disinfection: ______________________

1. Is there only one entrance to the farm, which is the hygiene lock?

( )Yes ( ) No

1. The farmers and visitors wash their hands with soap before entering the farm?

( )Yes ( ) No

1. The farmers and visitors wash and disinfect their hands before entering the farm?

( )Yes ( ) No

1. Is there clean towel/disposable towel?

( )Yes ( ) No

1. Is here clean boots/shoe covers and overalls/disposable protective clothes available in the lock?

( )Yes ( ) No

1. The farmer and co-workers change their “normal” clothes and use dedicated boots and clothes before entering the farm?

( )Yes ( ) No

1. Farm lay out

**Part 3 Animal antibiotic use in farm**

85. Did you use animal antibiotic or other drugs during the last 6 months?

( ) Yes ( ) No

86.If yes, please list for each different antibiotic separately:

1. Name: __________why: __________ animals:__________how many:_______

how long:_______how often: __________dose per animal :__________

1. Name: __________why: __________ animals:__________how many:_______

how long:_______how often: __________dose per animal :__________

1. Name: __________why: __________ animals:__________how many:_______

how long:_______how often: __________dose per animal :__________

1. Name: __________why: __________ animals:__________how many:_______

how long:_______how often: __________dose per animal :__________

87. If yes, by injection or with the food or the water?______________

88. Did you isolate ill animals? ( )Yes ( ) No

89. Do you ever come in contact with antibiotics that are given to pigs or chickens?

1□ Never

2□ Rarely

3□ Sometimes

4□ Often

5□ All the time

90.Do you ever come in direct contact with blood, urine, feces, tissue, fluids, etc. when working with in this farm?

1□ Never

2□ Rarely

3□ Sometimes

4□ Often

5□ All the time

Thank you for your participation in this project
