# Supplementary material for: A One Health approach to assessing occupational exposure to antimicrobial resistance in Thailand: The FarmResist project
Source: PLoS One. 2021 Jan 28;16(1):e0245250. doi: 10.1371/journal.pone.0245250 (PMC7842938; doi:10.1371/journal.pone.0245250)
Supplement: S2 File — (DOCX) [file pone.0245250.s002.docx]

**แบบสอบถาม**

**โครงการความเสี่ยงต่อการดื้อยาต้านจุลชีพจากการประกอบอาชีพในเกษตรกรเลี้ยงหมูและไก่**

**(The occupational risk of antimicrobial resistance among pig and poultry farmers)**

การศึกษานี้ศึกษาเป็นการศึกษาความเสี่ยงต่อการดื้อยาต้านจุลชีพจากการประกอบอาชีพในเกษตรกรเลี้ยงหมูและไก่ของจังหวัดน่าน มีวัตถุประสงค์เพื่อประเมินความชุกของการดื้อยาต้านจุลชีพ ศึกษาความสัมพันธ์ระหว่างการใช้ยาต้นจุลชีพกับปัจจัยการดื้อยาต้านจุลชีพในสัตว์เลี้ยงและเกษตรกร วิเคราะห์การสัมผัสเชื้อดื้อยาของเกษตรกในด้านชีวภาพและคุณลักษณะของฟาร์ม สร้างแบบประเมินความเสี่ยงจากการดื้อยาต้านจุลชีพในเกษตรกร และสร้างโมเดลประเมินความเสี่ยงต่อการดื้อยาต้านจุลชีพจากการประกอบอาชีพในเกษตรกรเลี้ยงหมูและไก่

*(การตอบแบบสอบถามชุดนี้จะใช้ระยะเวลาประมาณ 20-30 นาที/คน และผู้ถูกสัมภาษณ์มีสิทธิ์เลือกที่จะไม่ตอบข้อใดก็ได้)*

**ส่วนที่ 1 ลักษณะการทำงานและพฤติกรรมของเกษตรกร**

**1.1 ลักษณะทั่วไปของเกษตรกร**

1. เพศ : ( ) ชาย ( ) หญิง อายุ : ……………….. ปี
2. ส่วนสูง : ……………….. เซนติเมตร น้ำหนัก : ……………….. กิโลกรัม
3. บ้านเลขที่: …………………………………….. หมู่บ้าน : …………………………………….
4. ระดับการศึกษา:  ไม่ได้เรียนหนังสือ   ประถมศึกษา  มัธยมศึกษาตอนต้น

 มัธยมศึกษาตอนปลาย  ปวช./ปวส.  ปริญญาตรี  สูงกว่าปริญญาตรี

1. กิจกรรมยามว่าง (โปรดระบุ) : ……………………………………………………………………………………..
2. ท่านเดินทางไป หรืออาศัยอยู่ต่างประเทศในช่วง 12 เดือนที่ผ่านมาหรือไม่

 ใช่ ที่ใด.................................................และระยะเวลานานเท่าใด............................

 ไม่ใช่

1. อาชีพปัจจุบัน  เจ้าของฟาร์มปศุสัตว์  คนงานในฟาร์มปศุสัตว์  อื่นๆ (โปรดระบุ)…………………………….
2. คุณทำงานในฟาร์มเลี้ยงสัตว์มานานเท่าไหร่ ………….. ปี ……………..เดือน
3. หน้าที่ความรับผิดชอบของคุณในฟาร์ม …………………………………………………………………………………………...
4. ขั้นตอนการปฏิบัติงานของท่าน

| **ขั้นตอนการปฏิบัติงาน** | **คำอธิบาย** |
| --- | --- |
|  |  |
|  |  |
|  |  |
|  |  |

1. งานอื่นๆนอกเหนือจากงานหลัก (โปรดระบุ): ……………………………………………………………………………………….
2. ประวัติการทำงาน

| **ปี พ.ศ.** | **อาชีพและลักษณะงาน** |
| --- | --- |
|  |  |
|  |  |
|  |  |
|  |  |

**1.2 ลักษณะการทำงาน**

1. ท่านทำงานในคอกสัตว์เฉลี่ยวันละกี่ชั่วโมง (โปรดระบุ) ………………………………………………………ชั่วโมง
2. ท่านทำงานในฟาร์มสัตว์เฉลี่ยวันละกี่ชั่วโมง (โปรดระบุ) ………………………………………………………ชั่วโมง
3. ท่านสวมอุปกรณ์ป้องกันอันตรายระหว่างทำงานหรือไม่  ใช่  ไม่ใช่
4. โปรดระบุชนิดของอุปกรณ์ป้องกันอันตรายที่ท่านสวมขณะทำงาน

 ถุงมือ ชนิด...........................  หน้ากาก ชนิด...........................

 ชุดทำงาน ชนิด...........................  รองเท้า ชนิด...........................

 แว่นตา ชนิด...........................  อื่น ๆ (โปรดระบุ)..............................................................................

1. ท่านสวมอุปกรณ์ป้องกันนานแค่ไหน

 มากกว่าหรือเท่ากับ 50 เปอร์เซ็นของเวลาทำงานทั้งหมด

 น้อยกว่า 50 เปอร์เซ็นของเวลาทำงานทั้งหมด

1. ท่านและเพื่อนร่วมงานเปลี่ยนจากชุดทำงานปกติ เป็นชุดเฉพาะสำหรับเข้าฟาร์มสัตว์ใช่หรือไม่

 ใช่  ไม่ใช่ (หากตอบไม่ใช่ ข้ามไปตอบข้อที่ 20.)

1. ท่านถอดชุดทำงานก่อนเข้าบ้านหรือไม่  ใช่  ไม่ใช่
2. อากาศในคอกสัตว์มีฝุ่นหรือไม่  มีฝุ่นเยอะมาก  มีฝุ่นเล็กน้อย  ไม่มีฝุ่นเลย
3. ท่านสวมหน้ากากป้องกันฝุ่นระหว่างทำงานหรือไม่

 ใช่ สวมมากกว่าหรือเท่ากับ 50 เปอร์เซ็นของเวลาทำงาน  ใช่ สวมน้อยกว่า 50 เปอร์เซ็นของเวลาทำงาน

 ไม่สวมเลย

1. ท่านล้างมือหลังจากใช้อุปกรณ์ป้องกันอันตรายหรือไม่  ใช่  ไม่ใช่
2. ท่านและผู้มาเยี่ยม ล้างมือด้วยสบู่ก่อนเข้าไปในฟาร์มสัตว์ใช่หรือไม่  ใช่  ไม่ใช่
3. ท่านและผู้มาเยี่ยม ล้างมือและฆ่าเชื้อก่อนเข้าไปในฟาร์มสัตว์ใช่หรือไม่  ใช่  ไม่ใช่
4. ท่านมีผ้าสะอาดสำหรับเช็ดมือหลังล้างมือก่อนเข้าฟาร์มสัตว์ใช่หรือไม่  ใช่  ไม่ใช่
5. ท่านมีรองเท้าสะอาด หรือที่คลุมรองเท้า หรือชุดป้องกันก่อนเข้าไปในฟาร์มสัตว์ใช่หรือไม่

 ใช่  ไม่ใช่

**1.4 ข้อมูลด้านสุขภาพ**

1. ท่านเป็นโรคเบาหวานหรือไม่  ใช่  ไม่ใช่  ไม่ทราบ
2. ท่านเป็นโรคเกี่ยวกับไตหรือไม่  ใช่  ไม่ใช่  ไม่ทราบ
3. ท่านเป็นโรคเกี่ยวกับตับหรือไม่  ใช่  ไม่ใช่  ไม่ทราบ
4. ท่านเป็นโรคผิวหนัง (เช่น ผื่นแพ้ สะเก็ดเงิน) หรือไม่  ใช่  ไม่ใช่  ไม่ทราบ
5. ท่านป่วยด้วยโรคระบบทางเดินอาหารหรือไม่  ใช่  ไม่ใช่  ไม่ทราบ
6. ท่านเป็นโรคภูมิแพ้ และ/หรือ แพ้อาหารหรือไม่  ใช่ โปรดระบุสิ่งที่ท่านแพ้.......................................

 ไม่ใช่  ไม่ทราบ

1. ท่านมีโรคประจำตัวอื่นๆ ใช่หรือไม่  ใช่โปรดระบุโรคประจำตัวของท่าน...........................

 ไม่ใช่  ไม่ทราบ

1. ในช่วง 6 เดือนที่ผ่านมา ท่านใช้ยาปฏิชีวนะ (แก้อักเสบ) หรือไม่

 ใช่  ไม่ใช่ (หากตอบไม่ใช่ ข้ามไปตอบข้อที่ 39.)

1. โปรดระบุชนิดยา.............................................................................................
2. โปรดระบุปริมาณยาปฏิชีวนะที่ท่านใช้............................................................
3. ท่านใช้ยาปฏิชีวนะบ่อยเพียงใด  สัปดาห์ละครั้ง  เดือนละครั้ง  ปีละครั้ง  หลายๆ ปีครั้ง
4. เหตุผลที่เลือกใช้ยาปฏิชีวนะ (ยาแก้อักเสบ)..........................................................................................................

..............................................................................................................................................................................

1. ในระยะเวลา 6 เดือนที่ผ่านมาท่านทำงานในสถานพยาบาลหรือไม่  ใช่  ไม่ใช่  ไม่ทราบ
2. ในระยะเวลา 6 เดือนที่ผ่านมาท่านเคยเข้าพักรักษาตัวในโรงพยาบาลหรือไม่  ใช่  ไม่ใช่  ไม่ทราบ
3. ในระยะเวลา 6 เดือนที่ผ่านมาท่านป่วยด้วยโรคระบบทางเดินอาหารหรือไม่  ใช่  ไม่ใช่  ไม่ทราบ
4. ในระยะเวลา 6 เดือนที่ผ่านมาสมาชิกในครอบครัวท่านป่วยด้วยโรคระบบทางเดินอาหารหรือไม่

 ใช่  ไม่ใช่  ไม่ทราบ

1. ในระยะเวลา 6 เดือนที่ผ่านมาท่านเคยรักษาตัวที่โรงพยาบาลด้วยอาการบาดเจ็บจากสัตว์หรือไม่

 ใช่  ไม่ใช่  ไม่ทราบ

1. ท่านสูบบุหรี่หรือไม่  สูบ  ไม่สูบ (หากตอบไม่สูบ ข้ามไปตอบข้อที่ 50.)
2. ปัจจุบันท่านสูบบุหรี่จำนวนกี่มวนต่อวัน ...........................มวน/วัน
3. ท่านสูบบุหรี่ระหว่างทำงานในคอกสัตว์หรือไม่  ใช่  ไม่ใช่
4. ท่านล้างมือก่อนสูบบุหรี่หรือไม่  ล้างสมํ่าเสมอ  ล้างบางครั้ง  ล้างนานๆ ครั้ง  ไม่เคยล้าง
5. ท่านเคยสูบบุหรี่แต่หยุดไปแล้วใช่หรือไม่  ใช่ เคยสูบวันละ............................มวน/วัน  ไม่ใช่
6. ท่านหยุดสูบบุหรี่มาแล้วเป็นเวลานานเท่าใด ..................เดือน..................ปี
7. พฤติกรรมการบริโภคของท่านเป็นแบบใด

 ทานเจ ไม่มีเนื้อสัตว์หรือผลิตผลจากสัตว์เลย  มังสวิรัติ (ทานไข่ได้)

 ทานเฉพาะเนื้อสัตว์อย่างเดียว  ทานรวมทั้งผักและเนื้อสัตว์

1. เนื้อสัตว์ที่นำมาประกอบอาหารตามปกติได้มาโดยวิธีใด

 หาเองตามธรรมชาติ/ป่าชุมชน  ซื้อจากเพื่อนบ้าน/คนที่ไปหามา

 ตลาดสด/แหล่งขายเนื้อสัตว์  สัตว์ในฟาร์มของท่านเอง

 อื่นๆ .....................................................................................

1. ผักที่นำมาประกอบอาหารตามปกติได้มาโดยวิธีใด

 หาเองตามธรรมชาติ/ป่าชุมชน  ผักที่ท่านปลูกทานเอง

 ตลาดสด/แหล่งขายผัก  อื่นๆ ...............................................

1. ท่านปลูกผักใกล้ฟาร์มเลี้ยงสัตว์ใช่หรือไม่  ใช่  ไม่ใช่
2. นํ้าสำหรับการดื่มกินและบริโภคในครัวเรือนของท่านมีที่มาจากแหล่งใด

 นํ้าประปา  นํ้าจากแหล่งนํ้าธรรมชาติ เช่น ห้วย หนอง คลอง บึง

 นํ้าจากภูเขา (ประปาภูเขา)  อื่นๆ.....................................................................................

1. ในระยะเวลา 6 เดือนที่ผ่านมา ท่านเคยดื่มนํ้าโดยตรงจากแหล่งนํ้าธรรมชาติที่ไม่ผ่านการกรองหรือต้มให้สุกหรือไม่

 เคย  ไม่เคย

**1.5 ข้อมูลด้านสุขลักษณะ**

1. ท่านล้างมือ**ก่อน**รับประทานอาหารหรือไม่

 ใช่ ล้างทุกครั้ง  ใช่ ล้างบางครั้ง  ใช่ ล้างนาน ๆ ครั้ง  ไม่เคยล้างเลย

1. ท่านล้างมือ**หลัง**รับประทานอาหารหรือไม่

 ใช่ ล้างทุกครั้ง  ใช่ ล้างบางครั้ง  ใช่ ล้างนาน ๆ ครั้ง  ไม่เคยล้างเลย

1. ท่านรับประทานอาหารหรือดื่มน้ำในคอกสัตว์ใช่หรือไม่  ใช่  ไม่ใช่
2. ท่านอาบน้ำทุกครั้งหลังเลิกงานจากฟาร์มสัตว์ใช่หรือไม่  ใช่  ไม่ใช่
3. ในระยะเวลา 12 เดือนที่ผ่านมา ท่านเคยสัมผัสกับสัตว์ป่าใช่หรือไม่

 ใช่  ไม่ใช่ (หากตอบไม่ใช่ ข้ามไปตอบข้อที่ 62.)

1. โปรดระบุชนิดของสัตว์ป่าที่ท่านสัมผัส ……………………………………………………………………….
2. ในระยะเวลา 12 เดือนที่ผ่านมา ท่านเคยสัมผัสกับสัตว์เหล่านี้นอกฟาร์มของท่านหรือไม่

□ ไก่ □ หมู

□ หนู □ อื่นๆ โปรดระบุ ……………………………………………………………………

**ส่วนที่ 2 ลักษณะฟาร์ม**

1. โปรดระบุจำนวนหมูหรือไก่ในฟาร์มของท่าน

 สุกรตัวเมีย จำนวน …………… ตัว  ไก่พื้นเมือง จำนวน..............ตัว

 ลูกสุกรระยะหย่านม จำนวน.............ตัว  ไก่ชน จำนวน..............ตัว

 สุกรขุน จำนวน..............ตัว  ไก่พันธุ์เนื้อ จำนวน..............ตัว

 ลูกสุกรแม่พันธุ์ จำนวน …………… ตัว  ไก่ไข่ จำนวน..............ตัว

 หมูป่า จำนวน.............ตัว  อื่นๆ (โปรดระบุ)..................................... จำนวน..............ตัว

1. สำหรับฟาร์มหมูขุน ลูกสุกรมาจากแม่พันธุ์ตัวเดียวหรือหลายตัว

 ตัวเดียว  หลายตัว

1. สำหรับฟาร์มหมูแม่พันธุ์ ท่านเพาะพันธุ์แม่สุกรมาเองหรือซื้อมาจากแหล่งอื่นภายนอก

 เจ้าของฟาร์มเพาะพันธุ์เอง  แหล่งอื่นภายนอก

1. สำหรับฟาร์มหมูแม่พันธุ์ แม่สุกรมาจากหมู่บ้านเดียวกันหรือมาจากหมู่บ้านอื่น ๆ

 หมู่บ้านเดียวกัน  หมู่บ้านอื่น

1. คนงานหนึ่งคนดูแลไก่หรือหมูจำนวนกี่ตัว ………………………….ตัวต่อคน
2. ในฟาร์มของท่านมีสัตว์ชนิดอื่นอีกหรือไม่

 มี  ไม่มี (หากตอบไม่มี ข้ามไปตอบข้อที่ 70.)

1. โปรดระบุชนิดสัตว์อื่นๆในฟาร์มของท่าน

 โค/กระบือ จำนวน...........ตัว  แพะ/แกะ จำนวน............ตัว

 เป็ด จำนวน..............ตัว  อื่นๆ ............................................

1. ท่านมีสัตว์เลี้ยงหรือไม่ (เช่น แมว สุนัข กระต่าย เป็นต้น)

 มี  ไม่มี (หากตอบไม่มี ข้ามไปตอบข้อที่ 74.)

1. จากข้อ 70. ท่านมีสัตว์เลี้ยงทั้งหมดกี่ตัว (โปรดระบุ) ……………………ตัว
2. ชนิดสัตว์เลี้ยงของท่านคืออะไร (โปรดระบุ)

 สุนัข จำนวน............ตัว  แมว จำนวน..............ตัว

 นก จำนวน..............ตัว  กระต่าย จำนวน..............ตัว

 อื่น ๆ (โปรดระบุ).................................................................................

1. สุนัขหรือแมวสามารถเข้าไปในคอกสัตว์ได้หรือไม่  ได้  ไม่ได้
2. คอกสัตว์ในฟาร์มของท่านเป็นระบบเปิดหรือระบบปิด

 ระบบเปิด (ไม่ปิดทึบ อากาศถ่ายเทสะดวก)  ระบบปิด

1. โปรดระบุชนิดอาหารสัตว์ที่ท่านใช้

 อาหารสัตว์โดยเฉพาะ  อาหารคน

 อาหารสัตว์และอาหารคนผสมกัน  อื่นๆ (โปรดระบุ) ………………………………………………………………………

1. ฟาร์มของท่านใช้ระบบการเลียงแบบเข้า - ออกหมด ( ALL IN / ALL OUT)ใช่หรือไม่  ใช่  ไม่ใช่
2. ท่านขับไล่หนูในฟาร์มของท่านใช่หรือไม่  มี  ไม่มี
3. ท่านมีระบบการจัดการน้ำเสียในฟาร์มของหรือไม่

 มี  ไม่มี (หากตอบไม่มี ข้ามไปข้อที่ 81.)

1. ท่านมีระบบการจัดการน้ำเสียอย่างไร ……………………………………………………………………………………………………
2. ท่านทิ้งน้ำเสียจากฟาร์มของท่านไปที่ใด

 แหล่งน้ำธรรมชาติ (แม่น้ำ คลอง บึง ห้วย)  เก็บไว้เพื่อนำกลับมาใช้ในฟาร์มใหม่

 ใช้รดน้ำต้นไม้หรือพืชผักอื่นๆ  อื่นๆ (โปรดระบุ) ………………………………………………

1. ท่านทำความสะอาดคอกสัตว์ใช่หรือไม่  ใช่  ไม่ใช่ (หากตอบไม่ใช่ ข้ามไปตอบข้อที่ 83.)
2. ท่านทำความสะอาดบ่อยแค่ไหน

 ทุกวัน  สัปดาห์ละ 3-4 ครั้ง  สัปดาห์ละ 1-2 ครั้ง  อื่นๆ (โปรดระบุ) …………………

1. ท่านฆ่าเชื้อโรคในฟาร์มสัตว์ของท่านหรือไม่  ใช่  ไม่ใช่ (หากตอบไม่ใช่ ข้ามไปตอบข้อที่ 87.)
2. ท่านฆ่าเชื้อโรคในฟาร์มสัตว์ของท่านบ่อยแค่ไหน

 ทุกวัน  สัปดาห์ละ 3-4 ครั้ง  สัปดาห์ละ 1-2 ครั้ง

 เดือนละ 2 ครั้ง  เดือนละ 1 ครั้ง  อื่นๆ (โปรดระบุ) …………………

1. หลังจากทำความสะอาดหรือฆ่าเชื้อโรค ท่านปล่อยให้ฟาร์มแห้งก่อนใช้งานหรือก่อนนำสัตว์เข้าไปในในฟาร์มอย่างน้อย 24 ชั่วโมงใช่หรือไม่

 ใช่  ไม่ใช่

1. โปรดระบุชนิดของสารทำความสะอาดและสารฆ่าเชื้อในฟาร์มของท่าน

| ลำดับที่ | ชื่อผลิตภัณฑ์ |
| --- | --- |
|  |  |
|  |  |
|  |  |
|  |  |
|  |  |

1. ฟาร์มของท่านมีอ่างสำหรับล้างรองเท้าหรือล้างเท้าหรือไม่  มี  ไม่มี(หากตอบไม่มี ข้ามไปข้อที่ 94.)
2. ท่านใช้อ่างดังกล่าวเป็นประจำใช่หรือไม่  ใช่  ไม่ใช่
3. ท่านใส่นำยาฆ่าเชื้อลงไปในอ่างล้างเท้าหรือไม่  ใส่  ไม่ใส่(หากตอบไม่มี ข้ามไปข้อที่ 94.)
4. โปรดระบุชื่อผลิตภัณฑ์น้ำยาฆ่าเชื้อที่ใส่ลงไปในอ่างล้างเท้า …………………………………………………………….
5. โปรดระบุความเข้มข้นของน้ำยาฆ่าเชื้อในอ่างล้างเท้า …………………………………………………………………….
6. ท่านเปลี่ยนน้ำยาฆ่าเชื้อในอ่างล้างเท้าบ่อยแค่ไหน

 ทุกวัน  สัปดาห์ละ 3-4 ครั้ง  สัปดาห์ละ 1-2 ครั้ง

 เดือนละ 2 ครั้ง  เดือนละ 1 ครั้ง  อื่นๆ (โปรดระบุ) …………………

1. มีทางเข้าออกฟาร์มเพียงทางเดียวใช่หรือไม่  ใช่  ไม่ใช่
2. โปรดวาดแผนผังของฟาร์มคร่าวๆ (ระบุตำแหน่งบ้าน ฟาร์มสัตว์ ห้องเก็บอาหาร ที่บำบัดน้ำเสีย ที่ทิ้งน้ำเสีย อ่างล้างมือ อ่างล้างเท้า แหล่งน้ำธรรมชาติ)

**ส่วนที่ 3 การใช้ยาปฏิชีวนะในสัตว์**

1. ในช่วง 6 เดือนที่ผ่านมา ท่านใช้ยาปฏิชีวนะในสัตว์หรือไม่

 ใช่  ไม่ใช่ (หากตอบไม่ใช่ ข้ามไปตอบข้อที่100.)

1. โปรดระบุรายละเอียดการใช้ยาปฏิชีวนะต่อไปนี้

| ลำดับที่ | ชื่อยา | เหตุผลที่ใช้ | ชนิดสัตว์ | ปริมาณที่ใช้ทั้งหมด | ระยะเวลาที่ใช้ | ความบ่อย | ปริมาณที่ใช้ต่อสัตว์แต่ละตัว |
| --- | --- | --- | --- | --- | --- | --- | --- |
|  |  |  |  |  |  |  |  |
|  |  |  |  |  |  |  |  |
|  |  |  |  |  |  |  |  |
|  |  |  |  |  |  |  |  |
|  |  |  |  |  |  |  |  |
|  |  |  |  |  |  |  |  |

1. ท่านให้ยาปฏิชีวนะกับสัตว์ด้วยวิธีใด

 ฉีดยา  กินยา  ผสมยาเข้ากับอาหาร

 ผสมยาเข้ากับน้ำ  อื่นๆ (โปรดระบุ) ……………………………………………………………………………………….

1. เมื่อมีสัตว์ในฟาร์มของท่านป่วย ท่านแยกสัตว์ตัวนั้นออกหรือไม่  แยก  ไม่แยก
2. ท่านเคยสัมผัสโดยตรงกับยาปฏิชีวนะที่ให้กับสัตว์ในฟาร์มของท่านหรือไม่

 ไม่เคยเลย  นานๆครั้ง  บางครั้ง

 บ่อยๆ  ตลอดเวลา  อื่นๆ (โปรดระบุ) …………………

1. ท่านเคยสัมผัสโดยตรงกับเลือด ปัสสาวะ อุจจาระ เนื้อเยื่อ หรือของเหลว ของสัตว์ขณะท่านปฏิบัติงานหรือไม่

 ไม่เคยเลย  นานๆครั้ง  บางครั้ง

 บ่อยๆ  ตลอดเวลา  อื่นๆ (โปรดระบุ) …………………

ขอบคุณท่านที่สละเวลาตอบแบบสัมภาษณ์และเข้าร่วมโครงการวิจัยนี้
